# The Impact of Storytelling about an Innovative and Sustainable Organic Beef Production System on Product Acceptance, Preference, and Satisfaction

**DOI:** 10.3390/foods13182940

**Published:** 2024-09-17

**Authors:** Beata Ewa Najdek, Nora Chaaban, Margrethe Therkildsen, Barbara Vad Andersen

**Affiliations:** 1 Food Quality Perception and Society Team, iSense Lab, Department of Food Science, Aarhus University, 8200 Aarhus, Denmark; nora.chaaban@food.au.dk (N.C.); barbarav.andersen@food.au.dk (B.V.A.); 2 Differentiated and Biofunctional Foods Team, Department of Food Science, Aarhus University, 8200 Aarhus, Denmark; margrethe.therkildsen@food.au.dk

**Keywords:** sustainability, organic red meat production, storytelling, consumer acceptance, consumer satisfaction, consumer preference, eating habits

## Abstract

Food labels and storytelling are marketing tools used by the food industry to highlight and communicate important product characteristics to consumers. By using these tools, food companies can influence consumers’ attitudes toward the product and potentially the likelihood of purchase. In the present study, we investigated how storytelling about an innovative and sustainable organic beef production system influenced participants’ preference and acceptance of a veal steak product and, further, if some information characteristics were more important than others for consumer satisfaction. Without being aware that the samples were identical, participants (*n* = 224) tasted two veal steak samples: one steak sample was presented with information about the production system, and the other without information. Results showed that when the steak sample was presented with product information, compared to without information, it received significantly higher hedonic ratings (overall liking, liking of flavor, and liking of texture). This was likewise reflected in a greater preference for the steak sample when presented with product information. Furthermore, product information was found to positively impact the participants’ satisfaction with the steak sample regardless of their preference. Overall, our results suggest that the use of storytelling about the innovative and sustainable product system for veal steaks can positively influence consumers’ attitudes toward the product.

## 1. Introduction

Since the 1960s, the consumption of red meat has been increasing worldwide [1]. Although red meat is a great source of high biological value protein (i.e., it contains all the 10 essential amino acids) and essential nutrients that are beneficial for human health [2], previous research has shown associations between the consumption of red meat and potentially greater risk for developing cardiovascular diseases, type 2 diabetes, dementia, and colorectal cancer [1,2,3,4]. Not only is the intake of red meat associated with health issues but the growing demand for red meat has also led to several negative consequences on the environment, such as contributing to high levels of anthropogenic Greenhouse gas emissions (GHG), causing global warming [1,3,5]. Due to the negative impact on both health and the environment, the excessive intake of red meat has become a global concern. Therefore, identifying strategies for facilitating more sustainable consumer eating behaviors and thereby reducing the demand for red meat can not only have a positive impact on human health but also lead to significant improvements regarding the environment [3].

Changing consumer eating behaviors can be challenging, as eating behaviors are influenced by several factors such as taste and quality preferences, traditions, ethical or religious beliefs, societal norms, etc. [6,7]. Consumer quality perception is crucial for the survival of food products in the market, as perceived quality can affect both purchase decisions and experienced hedonic responses [8,9]. In the beef industry, experienced quality is often the primary factor important for consumer satisfaction and repeated purchases. This experienced quality is often linked to intrinsic sensory cues such as tenderness, juiciness, and flavor, which are important drivers of palatability, consumer acceptance, and satisfaction [8,10,11].

Although the experienced quality is responsible for consumer satisfaction, quality expectations at the time of purchase are often formed by extrinsic quality cues such as brand, labeling, country of origin, price, packaging, and production method [8,9,12]. The presence of labels on food products is an essential marketing communication tool used by food companies to attract consumers and influence their purchase decisions and, thereby, their food choices [13,14]. Food labels provide information about certain food product characteristics and can help direct consumers toward choosing food that matches their values [14]. Recent research has shown that food labels can further influence consumers’ expectations of a product, perception, and hedonic ratings of the product [15,16,17,18]. In a study by Lee et al. (2013), it was found that participants perceived foods labeled as “organic” more positively (i.e., fewer calories and better nutritional evaluations) compared to their counterpart, which was labeled as “regular” [17].

Storytelling is another marketing tool that has become more and more popular in agricultural marketing over the years [19]. Via storytelling, farmers can appeal to and catch consumers’ attention and inspire changes in consumption behavior [19]. According to Cardello and co-workers, perceptions of foods are guided by expectations developed during previous exposures and current available information [20,21,22]. Thus, expectations affect the ways in which consumers appreciate foods.

Consumers are known to express some degree of pleasure with a stimulus that corresponds to their expected pleasure with that stimulus. This takes place mainly via assimilation processes, where the perception of the stimuli is similar to the expectation. The “affective expectation model” posits that the degree of pleasure is formed based on a comparison to expectations of the stimuli, such that the expectation often determines the hedonic reaction [23]. As such, the more consumers expect to like, e.g., foods and drinks [24], the more they like them once the food and drink are experienced.

Access to product information, specifically, has been found to add hedonic value to the eating experience for some people [25]. Information about the origin is hypothesized to affect a consumer’s evaluation in two ways: either as a quality cue by hinting to other characteristics, such as sensory characteristics, or by their symbolic role, i.e., ethical values, authenticity, or the ability to awaken memories of past experiences [26]. In line with this, access to product information was mentioned to be important for several participants in the focus group by Andersen and Hyldig [27]. This study found that product information could be a source of satisfaction by bringing knowledge about the food’s history, e.g., origin, production method and animal welfare, and healthiness, e.g., via information about ingredients. On a more general level, the importance of knowing about food’s history for satisfaction can be related to the fact that this provides insights into the food’s production chain and allows the consumers to make choices reflecting their personal values, e.g., values around organic production [24]. Yet, the use of storytelling in concrete food-related studies is limited, and therefore, more research is needed to study how storytelling can influence consumers’ quality perception of food and possibly lead to changes in food behaviors.

A new, more sustainable, and organic beef production system was developed in relation to the present study. The aim of this study was to investigate how storytelling about the innovative and sustainable organic beef production system influenced participants’ preference and acceptance of a veal steak product and whether some information characteristics were more important for consumer satisfaction than others.

## 2. Materials and Methods

### 2.1. Overall Study Design

The study followed a randomized cross-over design, where each participant, in randomized order, consumed and evaluated two identical veal steak samples, one with and one without product information, respectively. Note: the participants were not told that the samples were identical.

### 2.2. Participants and Recruitment

The study was carried out in the fall (2022) as part of a Food Festival in Aarhus, Denmark. The Food Festival is an outdoor event where visitors can explore food-related topics through tasting and discussion activities with professionals across the food chain. The study was conducted over a two-day period in a tent affiliated with the Department of Food Science at Aarhus University. Visitors to the tent were welcomed and invited to participate in the research study without revealing the aim of the study. Inclusion criteria were Danish-speaking adults (above 18 years) who wanted to taste veal steak samples and answered questions related to the experiment via an online questionnaire. In total, 476 visitors participated in the experiment. Due to incomplete responses, replies from 376 participants were included in the analysis of preference, acceptance, and satisfaction ratings. Analyses of participant characteristics and personal drivers were analyzed based on replies from 224 of these participants. Characteristics of participants are summarized in Table 1.

### 2.3. Steak Samples

Samples from veal strip loin filets (M. logissimus lumborum) from Jersey (dairy cows) × Simmental (beef cattle) cross-bred heifer calves were used. Calves were raised with a nurse cow and put on a grass-based diet until slaughtering at the age of 12–16 months. Steaks (2 cm width) were sliced from the filets, vacuum-packed in pairs, and kept in the fridge until frying during the experiment days.

### 2.4. Product Information

Each participant received two identical steak samples with different information. The fact that the samples were identical was not revealed to the participants. For one of the samples (steak sample with product information), information about the characteristics of the innovative sustainable organic production system was presented (Table 2). For the second steak sample (steak sample without product information), only information about the product type was presented. No further details about the sample were given.

### 2.5. Procedure

Prior to the first day of the study, veal filets were sliced into steaks 2 cm wide and vacuum-packed in pairs. The vacuum-packed steaks were then divided into two groups: one group where the steak samples were presented without product information and a second group where the steak samples were presented with product information. Steaks were then stored in a fridge at 4 °C until frying on the day of the study.

On the study day, steaks were fried on a continuous basis as visitors volunteered for the study to ensure they all experienced warm and freshly prepared samples. The frying took place on-site and further served to attract visitors to the tent. To avoid participants identifying the samples as identical, steaks from the two groups were fried on separate frying pans, cut on separate cutting boards, and presented in containers on separate plates—in general, kept separate at all times during the study. Frying followed a standardized procedure: a few drops of grape-seed oil were heated in a pan. When warm, steaks from each group were placed on their separate frying pan and flipped every two minutes until reaching a core temperature of 63 °C. Steaks were then removed from the frying pan, sliced into bite-size pieces, and put in transparent containers with a fork. Steak samples were placed on separate plates with signs describing the two types of production system characteristics, as specified in Table 2.

Before taking part in the experiment, visitors were informed that the aim of the study was to collect data about their opinions on veal steak samples after tasting. Furthermore, participants were informed about the legal basis of the study, data treatment and protection, and their rights as research participants and gave written consent. The participants conducted the study by following a step-wise procedure in an online questionnaire, which they accessed via their mobile phones. Once completed, participants were thanked for their participation.

### 2.6. Questionnaire

First, participants were asked to choose which of the two samples they would like to taste first (facilitated order randomization). After tasting, participants were instructed to rate their overall liking of the sample, as well as their liking of the flavor and the texture. Ratings were collected on a 15 cm Visual Analogue Scale (VAS) with the anchors “Not at all” (0 cm) to “Extremely” (15 cm). Once both steak samples were evaluated, participants were asked to indicate which of the two steak samples they preferred. Participants were then presented with each of the four product information characteristics (Table 2) and asked to rate the extent to which each of the characteristics influenced their preference and satisfaction with the steak sample. Lastly, participants were asked questions about their habitual red meat consumption, aspects influencing red meat purchase, personal drivers to food-related pleasure in general, and demographics, i.e., gender, age, height, and weight.

### 2.7. Statistical Analyses

A paired T-test was used to study the effect of product information on acceptance (overall liking, liking of flavor, and texture) by comparing VAS ratings between the two steak samples, with and without product information.

A two-way ANOVA was conducted to study the impact of product information on preference and satisfaction. The model included two main factors: choice behavior (i.e., preferred steak sample; ‘with product information’, ‘without product information’ or ‘no preference’) and product information (‘characteristic 1’, ‘characteristic 2’, ‘characteristic 3’, ‘characteristic 4’, and ‘all characteristics’) as well as their interaction. Furthermore, two-way ANOVA was conducted to study the significant differences in the importance of six different drivers to food-related pleasure within the three groups (based on preferred steak samples). This model included two main factors: choice behavior (i.e., preferred steak sample; ‘with product information’, ‘without product information’ or ‘no preference’) and food-pleasure drivers (‘sensory characteristics’, ‘collative characteristics’, ‘post-ingestive sensations’, ‘cognitive aspects’, ‘product information’ and ‘eating context’), as well as their interaction. Tukey’s post hoc analyses were used to specify significant differences among pairs in the independent variables (satisfaction, preference, and importance for food-related pleasure) within and between groups. Furthermore, one-way ANOVA was conducted to study significant differences between groups and the importance of product information for food-related pleasure.

Descriptive statistics were used to analyze consumption frequency data, purchase characteristics, choice behavior (i.e., preferred steak sample), and study mean/std ratings on VAS scales.

Paired *t*-test analyses were carried out in Microsoft Excel (version 2303), and two-way ANOVA and Tukey’s post hoc analyses were carried out in XLSTAT 2024.2.2 1422 (Addison, January 2020, New York, NY, USA). The significance level was set at α ≤ 0.05.

## 3. Results

### 3.1. The Influence of Product Information on Acceptance Ratings

**Figure 1:**
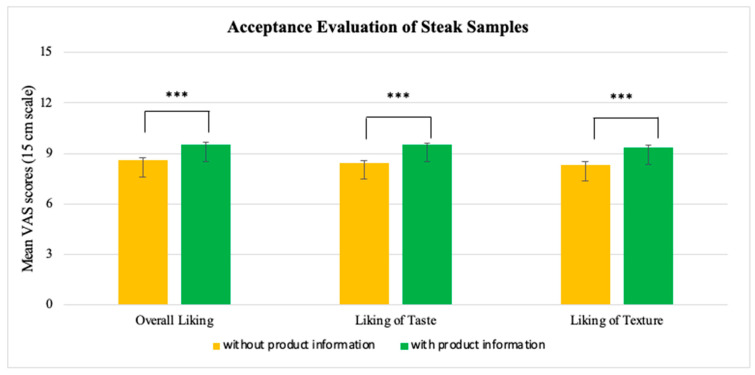
Mean VAS scores (15 cm scale) for overall liking, liking of taste, and liking of the texture of steak samples with and without product information (*n* = 376). Error bars are in standard error of mean (SEM). ***: *p* < 0.001.

Overall, acceptance ratings of veal steak samples, both with and without product information, were above neutral, indicating that the samples were generally liked (Figure 1).

For the steak sample without product information, ratings of overall liking, liking of taste, and liking of texture were 8.59, 8.84, and 8.31, respectively. For the steak sample with product information, ratings of overall liking, liking of taste, and liking of texture ratings were 9.59, 9.57, and 9.42, respectively. Ratings of steak samples with product information were significantly higher compared to steak samples without product information: overall liking (*p* < 0.001), liking of taste (*p* < 0.001), and liking of texture (*p* < 0.001).

### 3.2. Importance of Product Information for Preference

A total of 54% of the participants preferred the steak sample with product information, while 24% preferred the steak sample without product information, and 22% did not have a preference among the two samples (results not visualized).

**Figure 2:**
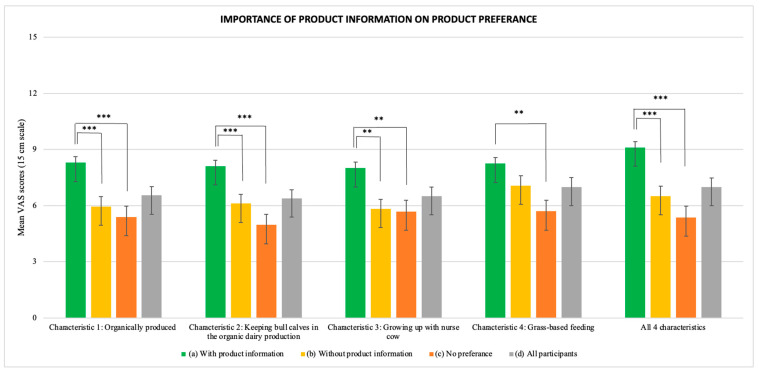
Mean VAS scores on the importance of product information characteristics among participants who preferred steak sample with product information (*n* = 205), participants who preferred steak sample without product information (*n* = 90), participants who did not have a preference (*n* = 81), and all participants (*n* = 376). **: *p* < 0.001 and ***: *p* < 0.0001.

When asked about the extent to which the four information characteristics of the production system (specified in Table 2) influenced their preference, mean importance ratings for the total group of participants were 7.25 for characteristic 1, ‘organically produced’, 7.09 for characteristic 2 ‘keeping bull calves in the organic dairy production’, 7.11 for characteristic 3 ‘grew up with nurse cow’, 7.53 for characteristic 4 ‘fed grass-based feed, and 7.84 for a combination of all four characteristics (Figure 2), indicating a close to neutral importance. Participants who preferred the steak sample with product information generally rated product information as significantly more important for preference than participants who preferred the steak sample without product information (*p* < 0.0001) and participants who did not have a preference (*p* < 0.0001) (Figure 2). No significant difference was found in ratings between participants who preferred steak samples without product information and participants who did not have a preference. The same trend was found for all four product information characteristics.

The mean importance of the product being ‘organically produced’ was 8.35 ± 3.72 among participants preferring the sample with product information, compared to 5.72 ± 3.99 among those preferring the sample without product information (*p* = 0.004) and 5.45 ± 4.20 among those showing no preference (*p* = 0.001). The mean importance of ‘keeping bull calves in the organic dairy production’ was 8.15 ± 3.76 among participants preferring steak sample with product information, compared to 5.84 ± 3.66 among those preferring sample without product information (*p* = 0.028) and 5.10 ± 3.82 among those showing no preference (*p* < 0.00001). The mean importance of the information of veal ‘growing up with nurse cow’ was 8.09 ± 4.01 among participants preferring steak samples with product information, compared to 5.54 ±3.81 among those preferring steak samples without product information (*p* = 0.006) and 5.75 ± 3.84 among those showing no preference (*p* = 0.035). The mean importance of veal being fed ‘grass-based feed’ was 8.37 ± 3.87 among participants preferring the sample with product information, compared to 6.70 ± 4.16 among the group preferring the sample without product information (ns) and 5.78 ± 4.19 among those showing no preference (*p* = 0.009). Lastly, when asked to make an overall evaluation of all four characteristics combined, the mean rating of importance was 9.22 ± 3.78 among participants preferring the sample with product information, compared to 6.16 ± 4.11 among participants preferring the sample without product information (*p* < 0.00001), and 5.28 ± 4.26 among participants showing no preference (*p* < 0.0001) (Figure 2).

### 3.3. Importance of Product Information for Satisfaction

**Figure 3:**
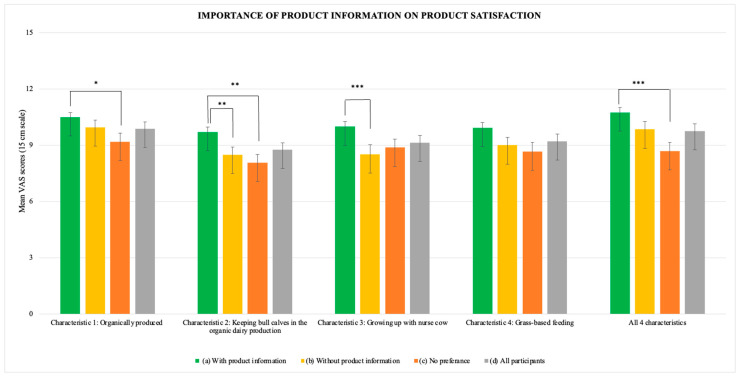
Mean VAS scores on the impact of product information on satisfaction among (a) participants who preferred steak sample with product information (*n* = 205), (b) participants who preferred steak sample without product information (*n* = 90), (c) participants who did not have a preference (*n* = 81), and (d) all participants (*n* = 376). *: 0.01 < *p* < 0.05, **: *p* < 0.001 and ***: *p* < 0.0001.

Participants were asked to rate the extent to which the four product information characteristics influenced overall satisfaction with the product (Figure 3). For the total group of participants, mean values for characteristic 1, ‘organically produced’, characteristic 2 ‘keeping bull calves in production’, characteristic 3, ‘grew up with nurse cow’, characteristic 4 ‘grass-based feed’, and a combination of all characteristics were 10.17, 9.14, 9.47, 9.50, and 10.21, respectively, indicating a positive impact on satisfaction.

Participants who preferred steak samples with product information generally showed significantly higher ratings than participants who preferred steak samples without product information (*p* < 0.0001) and participants without preference (*p* < 0.0001). No significant difference was found between participants preferring steak samples without product information and participants showing no preference. Specifically, the mean importance of the product being ‘organically produced’ was significantly more important for the participants who preferred the steak sample with information (10.47 ± 2.96) compared to the participants without a preference (9.32 ± 3.58) (*p* = 0.03). The mean importance of the characteristic ‘keeping bull calves in the organic dairy production’ was significantly higher among participants preferring steak samples with product information (9.72 ± 2.72) compared to those preferring samples without product information (8.41 ± 3.38, *p* = 0.032) and those without preference (8.07 ± 3.63, *p* = 0.005). The mean importance of the information of veal ‘growing up with nurse cow’ was 10.05 ± 3.13 among participants preferring steak samples with product information, compared to 8.51 ± 3.40 among those preferring steak samples without product information (*p* = 0.015) and 8.72 ± 3.76 among having no preference (not significantly different from either group). No significant difference was found for the characteristic ‘grass-based feed’. Lastly, participants were asked to evaluate the importance of all four characteristics combined for their satisfaction with the product, and the results are as follows. The mean rating of importance was 10.75 ± 2.69 among participants who preferred steak samples with information, compared to 8.87 ± 4.04 among participants showing no preference (*p* = 0.001) and 9.87 ± 3.33 among those preferring the sample without information (not significantly different from either group). These findings are illustrated in Figure 3.

### 3.4. Participants’ General Drivers to Food-Related Pleasure

**Figure 4:**
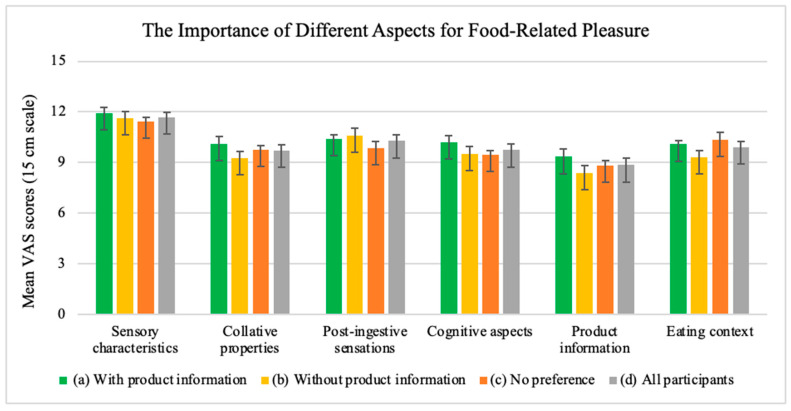
Mean VAS scores and SEM on the importance of six aspects of food-related pleasure among (a) participants who preferred the steak sample with product information (*n* = 138), (b) participants who preferred the steak sample without product information (*n* = 47), (c) participants who did not have a preference (*n* = 43), and (d) all participants (*n* = 228).

Participants were asked to rate the importance of six common aspects potentially driving their pleasure around food as shown in Figure 4.

For the total group of participants, sensory characteristics were significantly more important for food-related pleasure compared to all aspects: collative characteristics (*p* < 0.0001), post-ingesive sensations (*p* < 0.0001), cognitive aspects (*p* < 0.0001), product information (*p* < 0.0001), and eating context (*p* < 0.0001) as shown in Figure 4. Product information was significantly less important for food-related pleasure compared to all aspects: collative characteristics (*p* = 0.046), post-ingestive sensations (*p* < 0.0001), cognitive aspects (*p* = 0.028), and eating context (*p* = 0.004) as shown in Figure 4.

When analyzing the importance of each driver to pleasure within the three groups, i.e., those preferring the sample ‘without product information’, ‘with product information’, or ‘no preference’, respectively, significant differences were likewise found. For participants who preferred steak samples with product information, sensory characteristics were significantly more important than product information (*p* = 0.000) and eating context (*p* = 0.049). For participants who preferred steak samples without product information, the sensory characteristics were significantly more important than collative characteristics (*p* = 0.000), cognitive aspects (*p* = 0.003), product information (*p* < 0.0001), and eating context (*p* = 0.000). And, for the participants who did not have a preference, sensory characteristics were significantly more important than cognitive aspects (0.037) and product information (*p* < 0.0001), and eating context was significantly more important than post-ingestive sensations (*p* = 0.007). These findings are illustrated in Figure 4.

When comparing the three groups in terms of the importance of ‘sensory characteristics’, ‘collative properties’, ‘physical and mental sensations’, cognitive aspects’, ‘product information’, and ‘dining environment’, respectively, there were no significant differences between participants who preferred steak samples with product information and participants who preferred steak samples without product information (Figure 4).

## 4. Discussion

The main aim of the present study was to investigate how storytelling about a new, sustainable organic beef production system influenced consumers’ preferences and acceptance. Key results from the present study confirmed that product information influenced participants’ perception of the steak samples. This was found through more consumers preferring the steak sample when presented with product information. Also, presenting product information resulted in higher overall acceptance ratings and hedonic ratings of taste and texture.

The effect of food labeling on consumers’ perception and liking of food has been demonstrated in several studies previously [12,15,17]. In a study by Schouteten et al., (2015), it was demonstrated how different labeling (e.g., reduced salt, light, and light with reduced salt) on the same samples of cheese biased both the sensory expectations before tasting and the actual sensory perception after tasting [16]. Although the cheese samples were identical, participants expected the ‘reduced salt’ to have less salt intensity compared to the control and the ‘light’ to be less fatty than the control, which was likewise reflected in the perception after consumption. In the present study, an association between the product information characteristics and perception of sensory product characteristics seems to be created, both when the information hinted at sensory alterations (i.e., information characteristic 4) as well as when not hinting explicitly at altered sensory characteristics (i.e., characteristic 1–3). Similar results have been found previously. In a previous review by Fernqvist and Ekelund (2014), [12] it was found that different credence characteristics of food products, i.e., qualities that cannot be evaluated even after purchase, and therefore are dependent on information signaling such as food labels, can generate sensory expectations affecting consumer liking.

A secondary aim of this study was to investigate if certain information characteristics about the new, sustainable, organic production system were more important for consumer preference and satisfaction than other information characteristics. Although the presence of product information influenced consumer preference and satisfaction, no specific product information characteristic was found to be most important for the participants. Meaning that the characteristics of organically produced, keeping bull calves in production, growing up with nurse cows, and grass-based feed were equally important. A combination of all four characteristics was found to be important for consumer preference and satisfaction, but the combination did not prove superior to the importance of each characteristic individually. Risius and Hamm (2017) analyzed important attributes for consumers in their decision-making. The study found that consumers highly valued the attribute ‘organic’, followed by ‘extensive nurse cow’ and ‘pasture-based’ [28]. Another consumer study analyzed how information affected participants’ evaluations of lamb meat sourced from two different breeding systems: meat from lambs reared by stall-fed mothers and lambs reared by pasture-fed mothers. In the blind test, meat from lambs reared by stall-fed mothers received higher hedonic scores on tenderness, flavor, and juiciness compared to meat from lambs reared by pasture-fed mothers. However, when participants were presented with meat products labeled with information regarding the animal feeding system, meat from lambs raised by pasture-fed mothers was preferred over meat from lambs raised by stall-fed mothers [29]. This study shows the power of information in shifting consumer preferences.

It should be noted that as participants’ knowledge level about the current production system was not tested, it cannot be ruled out that their knowledge level (i.e., familiarity with the production system) influenced the results of the current study, just as it is unknown if the participants understood the deeper meaning of the information characteristics; organically produced, keeping bull calves in organic dairy production, growing up in a nurse cow system and grass-based feeding. It can be hypothesized that if the ordinary consumer (participating in the current study) has no concept of the currently used production system, the results are biased toward their concept of what the system might be and the implications of the novel system on aspects such as animal welfare, etc. The study by Conner et al., (2008a) points to the majority of consumers lacking knowledge about production practices [30], which can result in wrong pre-assumptions [31]. For example, it has been found that some consumers mistakenly assumed that all cattle were grass-fed [32], and therefore, they did not recognize the significance of keeping cattle on pasture [33].

The current study suggests that information characteristics, despite hinting at altered sensory characteristics or not, can be associated with higher quality aspects and flavor characteristics of the meat. From a scientific perspective, these results suggest that consumers may project their attitude on extrinsic product characteristics onto intrinsic perceptions (i.e., sensory perceptions), which as a result, affect their hedonic attitude (i.e., satisfaction and preference). Thus, the crucial importance of sensory characteristics in guiding affective ratings. The importance of sensory characteristics was further shown, as participants explicitly rated quality/flavor as the most important factor when buying red meat and rated sensory characteristics as the most important driver of food-related pleasure. These findings align with previous studies showing that intrinsic attributes such as color, marbling, flavor, juiciness, tenderness, freshness, and healthiness contribute to consumers’ perception of beef quality [8] and that sensory attributes, such as flavor, juiciness, tenderness, and aroma, are the main determinants of consumers’ acceptability and preference for meat products [34].

In summary, no matter the underlying mechanism guiding preference formation, the study showed that providing product information had a notable positive impact on participants’ preference and acceptance of the steak samples.

Lastly, while analyzing the results, it is important to acknowledge the limitations of this study. Firstly, the sample of participants was limited to the specific population visiting the Food Festival, who can be expected to be interested in food-related topics, and generalizability to other consumer segments should carried out with caution. Also, the study relied on self-reported ratings carried out in the specific context of the Food Festival, which may be subject to more disturbances than would be the case in a lab and potentially in-home setting. Thus, the ratings and evaluation of steak samples might not be completely comparable to other contexts.

## 5. Conclusions

The present study investigated how storytelling about an innovative and sustainable organic beef production system influenced participants’ preference and acceptance of a veal steak product, and further, if some information characteristics were more important than others for consumer preference and product satisfaction.

In conclusion, the findings provide confirmatory evidence that storytelling, in this case about the innovative and sustainable production system for veal steaks, can positively influence consumers’ preference and acceptance of the product. The majority of participants preferred the sample when presented with information, which was reflected in the hedonic ratings of overall liking, liking of taste, and texture of the samples. The effect of storytelling about the production system and its characteristics seemed to be mediated by the associations it created with the product’s sensory properties.

Additionally, all four information characteristics of the production system, i.e., ‘organically produced’, ‘keeping bull calves in production’, ‘grew up with nurse cow’, and ‘grass-based feed’ were important for consumer satisfaction and preference. No information characteristic was found to be more important than the other. Thus, all four information points can be considered positive storytelling elements.

## Figures and Tables

**Table 1:** Summary of participant characteristics.

Characteristics				No Preference
	General	Without Information	With Information	
**Gender** (% ‘males’/% ‘females’/% ‘other’)	48%/51%/1%	11.9%/9.7%	26%/35.5%	9.3%/8.8%
**Age** (years) ^a^	38.1 (±15.9)	37.1 (±16.3)	38.2 (± 16.5)	35.8 (±14)
**BMI** ^a^	24.4 (±5.0)	23.7 (±4.6)	24.6 (± 4.6)	25.3 (±5.5)
**General liking of red meat** ^a,b^	9.97 (±3.4)	10.31 (±3.6)	9.8 (± 3.4)	9.94 (±3.5)
**Frequency of red meat consumption** (5–7 days a week/1–4 days a week/Less than 1 day a week/Never)	2.8%/27.3%/29.8%/1.3%	1.3%/8.8%/12.1%/0.4%	2.9%/25.4%/29.6%/1.3%	0.4%/10.4%/7.1%/0.4%
**Purchase characteristics** (Price/Color/Organic/Quality/Animal welfare/Fat content/Tenderness/Other)	36.1%/22.9%/19.9%/39.6%/25%/17.7%/27.8%/3.5%	13.8%/7.1%/7.1%/15%/8.3%/6.7%/10.4%/1.3%	33.8%/23.8%/18.8%/37.9%/24.6%/16.7%/25.8%/3.8%	12.1%/7.1%/5.8%/11.7%/7.5%/5.4%/9.2%/1%

^a^ Mean (std). ^b^ Mean (std) rating collected on a 15 cm visual analog scale (VAS) anchored ‘not at all’ (0 cm) and ‘extremely’ (15 cm). *n* = 224.

**Table 2:** An overview of the product information presented with steak samples.

Steak Sample without Product Information	Steak Sample with Product Information
Steak from dairy bull calves	Steak from dairy bull calves
	**Characteristic 1:** Organically produced for the sake of the climate, animal welfare, and biodiversity.
	**Characteristic 2:** Produced from dairy bull calves to keep bull calves in organic meat production as dairy cows can deliver both milk and meat.
	**Characteristic 3:** Calves grew up with a nurse cow and received milk until the age of six months for the sake of animal welfare.
	**Characteristic 4:** Grass-based feed for the sake of natural cattle feed, healthy fatty acids, and meat flavor.

## Data Availability

The data presented in this study are available on request from the corresponding author. The data are not publicly available due to privacy restrictions.
